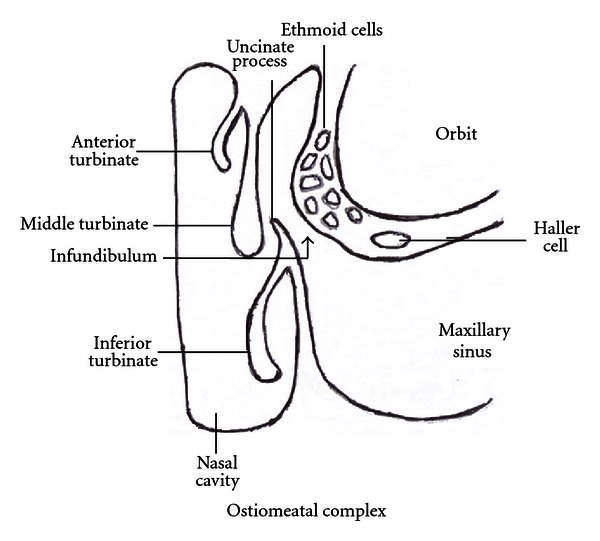# Rhinosinusitis in Children 

**DOI:** 10.5402/2012/851831

**Published:** 2012-12-05

**Authors:** Sukhbir K. Shahid

**Affiliations:** Department of Pediatrics and Neonatology, Shahid Clinic and Hospital, Maharashtra, Mumbai 400 077, India

## Abstract

Rhinosinusitis is the inflammation of the mucous membranes of nose and paranasal sinus(es). 5–13% of upper respiratory tract infections in children complicate into acute rhinosinusitis. Though not life threatening, it profoundly affects child's school performance and sleep pattern. If untreated, it could progress to chronic rhinosinusitis (CRS). The pathogens involved in perpetuation of CRS consist of multidrug-resistant mixed microflora. CRS is challenging to manage and could further extend to cause eye or intracranial complications. In children, CRS diagnosis is often either missed or incomprehensive. Due to this, morbidity and strain on healthcare budget are tremendous. Flexible fiberoptic endoscopy has revolutionized management of CRS. Its utility in children is being increasingly recognized. Optimal management entails specific appropriate antimicrobials as well as treatment of underlying causes. The aim is to normalize sinus anatomy and physiology and regain normal mucociliary function and clearance.

## 1. Introduction

Rhinosinusitis is a widely prevalent disease affecting more than 14% of adults and children [1–4]. It has high propensity to become chronic. Though the acute form of rhinosinusitis is unimicrobial, multiple microorganisms characterize the chronic form [5–7]. The latter microbes usually demonstrate antimicrobial resistance and pose a therapeutic challenge for the practising physician [8]. Fungi often coinhabit such chronically infected sinuses and are extremely difficult to eradicate [9]. They add on to the morbidities and complications [10, 11]. Maximum medical therapy often fails and surgical interventions become mandatory [12, 13]. This wells up healthcare costs. Hence early detection and prompt and appropriate treatment of rhinosinusitis could possibly avert CRS and its individual and societal burden [14, 15]. 

## 2. Definition

The combined term “Rhinosinusitis” was coined by 1997 Task Force of Rhinology and Paranasal Sinus Committee because sinusitis is invariably accompanied by rhinitis [16]. Acute rhinosinusitis implies sudden onset of two or more of the following symptoms: nasal discharge, stuffiness or congestion, facial pain/pressure, or anosmia/hyposmia [17, 18]. There may be associated fever, malaise, irritability, headache, toothache, or cough. When symptoms are present for 4–12 weeks, it is subacute rhinosinusitis. When they persist for more than 12 weeks, it is termed as “chronic rhinosinusitis” [19]. The latter results usually due to untreated/improperly treated/refractory acute rhinosinusitis. Recurrent rhinosinusitis is 4 or more episodes of acute sinus infection in one year with each episode lasting for about a week. Based on the etiology, rhinosinusitis could be viral, bacterial, fungal, parasitic, or mixed. 

## 3. Development and Anatomy of Paranasal Sinuses

Paranasal sinuses are air-filled hollows in the skull bones connected to the nose. Ethmoid and maxillary sinuses are present at birth and fully developed by 3 years. The development of sphenoidal sinus starts by 3 years and that of frontal sinus by 7 years; these are fully developed only by adolescence [20]. Sinuses have multiple functions; the most important of which is humidification and heating of inspired air, providing vocal resonance, lightening of skull bones, immune defence, and absorption of pressure variations [21, 22]. They are lined by mucous membrane made up of pseudostratified ciliated columnar epithelium with interspersed mucus-secreting goblet cells. This lining is in continuation with that of the nasal cavity. The sinus cavity is normally sterile. Its secreted mucus contains antimicrobicidal polypeptides and lipids which function as innate defence for the airways [23]. The continuous movements of the cilia towards the sinus orifice generate currents which clear the mucus from the sinus into the nasal cavity [24]. The main area of sinus drainage is the “ostiomeatal complex” present in the middle meatus on the lateral wall of the nasal cavity (Figure 1) [25]. Its borders and margins are ill-defined and it is more of a functional area for opening of the anterior ethmoid, maxillary, and frontal sinuses. It comprises maxillary ostia, infundibulum, uncinate process, hiatus semilunaris, ethmoid bulla, and middle meatus [26].

## 4. Etiopathogenesis

The integrity of the ostiomeatal complex is most crucial for sinus health. Ostial obstruction is usually the start point for sinusitis. It generates a negative pressure in the sinus, which leads to fluid seepage into the sinus. This fluid being a good culture media gets easily infected. This damages the lining cilia, and mucus production is increased. Mucociliary clearance thus gets compromised. A self-perpetuating cycle is established, which needs to be interrupted for optimal outcome [27]. One or more sinuses may be involved with infection. Isolated sphenoidal sinusitis is less common being seen in only 2.7% of sinus infections [28]. The “ostiomeatal complex” obstruction could be due to [29, 30]

1. anatomic abnormalities such as adenoidal hypertrophy, deviated nasal septum, concha bullosa, Haller cells, and so forth [31];
2. mucosal edema due to viral rhinitis and allergic rhinitis including aspirin sensitivity;
3. nonallergic rhinitis (vasomotor rhinitis, rhinitis medicamentosa, cocaine abuse);
4. nasal polyps;
5. unattended nasal foreign bodies;
6. immunodeficiency conditions (congenital or acquired), these comprise 1/3rd-2/3rd of cases of refractory chronic sinusitis [32];
7. cystic fibrosis;
8. ciliary dysfunction syndromes such as primary ciliary dyskinesia, Kartagener's syndrome, and so forth;
9. nasotracheal tube and prolonged ventilation;
10. prolonged nasogastric tube;
11. gastroesophageal reflux [33];
12. nasal tumours such as nasopharyngeal angiofibromas;
13. smoking: active and/or passive;
14. environmental pollution and irritants;
15. sarcoidosis;
16. Wegener's granulomatosis;
17. periodontitis/significant dental disease involving upper teeth which causes 5–10% of acute rhinosinusitis [34];
18. Hormonal (puberty, pregnancy, oral contraceptive use).

Swimming, diving, high-altitude climbing, and diabetes mellitus predispose to rhinosinusitis [29]. 

Viruses are most common causes of acute rhinosinusitis [35]. Within few days, bacterial invasion and proliferation set in. *Streptococcus pneumonia, Hemophilus influenzae, Moraxella catarrhalis, *beta-hemolytic* Streptococcus pyogenes* are usual pathogens cultured. *Mycoplasma and *chlamydial species have also been found associated with sinusitis in children [36–38]. With chronicity, polymicrobes supersede [39, 40]. These include staphylococci, alpha-hemolytic streptococci, anaerobes such as peptostreptococci,* Bacteroides *and* Fusobacterium *species, pseudomonads, other gram-negative enteric bacteria, and fungi. Dematiaceous fungi comprising of *Bipolaris, Curvularia, Exserohilum, Alternaria, Drechslera, Helminthosporium, Acremonium, Chrysosporium, Fusarium and Aspergillus* species are commonly isolated from CRS [41, 42]. *Paecilomyces lilacinus *is a rare cause of sinusitis in immunocompetent host [43]. *Trichoderma longibrachiatum* and *Ulocladium *have been recovered in some cases [44, 45]. Parasites as a cause of sinusitis are extremely rare and commonly encountered in those with immune disorders [46–49]. 

CRS is usually a consequence of untreated, improperly treated, or nonresponding acute rhinosinusitis. Entry of aerosolized environmental fungi into sinuses of children with allergic rhinitis could also initiate allergic fungal sinusitis (AFS). Fungal debris and thick, tenacious, and highly viscous allergic mucin are hallmarks of AFS. Allergic mucin is lamellated collection of inspissated inflammatory debris. These specific sinal secretions are either light brown to dark green in color and have been variously described as peanut butter or axle grease. In AFS, tissue invasion is absent, and marked eosinophilia with raised serum total IgE is noticed. It is an immunologically mediated disease with predominant Th2 type of immune response. The signature cytokines of this Th2 type of immune response, namely IL-4, IL-5, and IL-13, are raised in sinonasal fluids [50]. Researchers from Hopkins have found that the gene for acidic mammalian chitinase (AMCase) enzyme is more than 250 times active in CRS. This is assumed to be due to misplaced immunity to “ghost” organisms [51]. CRS also has rampant biofilm formation. Biofilms are structured, specialized, complex aggregates of adherent microorganisms encased in matrix or extracellular polymeric substance [52]. They are the sessile, metabolically inactive phenotype of bacteria/fungi which have adopted this state for survival. They are characterized by genetic diversity, structural heterogeneity, surface attachment, complex community interactions, and an extracellular matrix of polymeric substances. They cover more than 90% of the sinus mucosa in CRS. Adenoidal tissue, tonsils, otitis media, and cholesteatomas also demonstrate biofilms. These areas serve as nidus to propagate the virulent infection [53, 54]. Biofilms are multispecies community and this diversity helps organisms to gain a competitive advantage over antibiotics and host's defences. The germs continue to flourish in these biolayers and their close proximity in high numbers facilitates transfer of genetic material for antimicrobial resistance. This coupled with recurrent irrational antibiotic courses further promotes development of resistance. In many instances, in spite of *in vitro *sensitivity, *in vivo* resistance is seen. Biofilms thus serve as an effective survival strategy [55–57]. Microcolonies with well-developed channels to convey fluids and nutrients are seen in these biofilms [58]. Scanning electron microscopy (SEM), transmission electron microscopy (TEM), and confocal laser scanning microscopy (CLSM) with staining for live/dead organisms or fluorescent *in-situ* hybridization (FISH) are used to detect these biofilms [59–61]. Since sinus mucosa is directly adherent to bone, spread of infection to bones and osteitis is frequent; it is seen in 36–53% of cases of CRS in adults [62]. This could further extend to the adjacent skull, brain, or eyes [63]. 

Thus, CRS is predominantly a heterogeneous inflammatory disease. It is multifactorial with environmental and host general and anatomic factors all playing a role in its development. Persistence of infection (biofilms and osteitis), allergy, immunologic disorders, upper airway intrinsic factors, superantigens-induced polyclonal immune response, fungi with eosinophilic inflammation, remodeling, and metabolic problems such as aspirin sensitivity all contribute to the varied picture of chronic rhinosinusitis [64]. It is a tough disease to manage and demonstrates multiple recurrences. 

## 5. Clinical Features

Rhinosinusitis usually starts with an acute upper airway infection which persists beyond 7–10 days. The cold either does not improve, worsens after 7–10 days, or returns after an initial improvement (double sickening). It is also called “head cold.” It is associated with fever, malaise, irritability, facial pain or pressure, headache, cough, rhinorrhoea, nasal stuffiness, and diminution or loss of olfactory sensations. There may be postnasal drip, halitosis, and otitis media. With severe infection, purulent nasal secretions, high fever, and periorbital edema are seen [65]. Some may evolve into bacterial or persistent forms. However, the majority of acute infections resolve without antibiotics. Some of these resolved cases may recur as acute or subacute rhinosinusitis [66]. Anterior rhinoscopy usually reveals hypertrophied, red and inflamed inferior turbinate, nasal polyp, or pus at middle meatus. The latter can be better visualized with nasal endoscopy which may, however, be difficult in younger and uncooperative patients. This telescopic visualization can also detect adenoidal hypertrophy, foreign bodies, and other obstructions [67]. Eye or meningeal/brain infections may tag on with CRS. These polymicrobial infections have high morbidity and require urgent surgical interventions. 

Allergic fungal sinusitis comprises 5–10% of CRS. It is common in older immunocompetent children with no sex predilection. It differs from invasive fungal infections of paranasal sinuses of immunodeficient patients in its noninvasive nature, eosinophilia, and presence of allergic mucin. Features of AFS are similar to CRS except that pain is not common in the former. Also there are associated features of allergic rhinitis in AFS. AFS should be suspected when allergic rhinitis is recalcitrant. Proptosis, telecanthus, and malar flattening may be noticed in long-standing cases. Since proptosis is established over time, visual loss or diplopia are rarely associated. Children have commonly unilateral involvement in AFS compared to adults [68]. Sinusitis forms the initiating focus in 50% of the Henoch-Schonlein purpura (HSP) in children [69]. 

## 6. Diagnosis

Diagnosis of rhinosinusitis in children is not easy. It should be suspected when cold does not improve beyond 10 days or nasal stuffiness is present with purulent discharge, facial pain, headache, fever, and diminution/loss of sense of smell. Dental, ear, or eye problems, allergies, or environmental pollutants can produce similar symptoms and cause confusion. Sneezing and nasal itching are common with allergic rhinitis. In these children with isolated allergic rhinitis, purulent nasal discharge is usually not seen. In those children of allergic rhinitis with superadded sinusitis, the picture is altered, and along with nasal itching and sneezing, purulent nasal secretions and loss of olfaction sense may also be associated. Allergic rhinitis is not uncommonly seen associated with sinusitis; hence a search for triggering allergies needs to be performed in children presenting with rhinosinusitis. Anterior rhinoscopy and fibreoptic nasal endoscopy may be useful for accurate assessment of the middle meatus pathologies and detection of associated adenoiditis, nasal polyps, or nasal masses. This can also be used to collect specimen for testing secretions or mucosa. Patient cooperation is of utmost importance for these procedures. 

Transillumination of sinuses is useful in hands of experienced person. In this, a flashlight is placed against the patient's cheek and the doctor looks into the patient's open mouth. A lit-up reddened area is seen in the palatal area with normal sinuses. When sinuses are fluid-filled, this reddened area will not be visualized [70]. Near-infrared light (750–1100 nm) can penetrate deeper and permit enhanced illumination of deeper structures. But since this light is invisible normally, a charge-coupled device camera is used to capture and record images. This technique has been found to be a safe, reliable, low-cost, and simple aid for diagnosis of sinusitis [71]. 

Plain radiography of paranasal sinuses can be performed, but it has a limited diagnostic role. Water and Caldwell-Luc's views are taken for sinusitis. Haziness, opacification, or fluid level is suggestive of sinusitis [72]. CT scan of sinuses gives a better visualization and is a useful tool preoperatively. Limited axial and coronal cuts ordinarily suffice. Contrast is reserved for suspected suppuration. CT scan can pick up noninvasively ostiomeatal anomalies with great accuracy. Mucosal changes, intrasinus collections or growths, and adjacent bone changes can be visualized. Soft-tissue algorithms of CT scan reveal heterogeneity of signal intensity in AFS. Accumulations of heavy metals (iron, manganese, calcium) within the allergic mucin elicit enhanced signals. For orbital and intracranial extensions or in AFS, MRI is more informative. In AFS, allergic mucin is hyperdense on T1W1 images with signal void on T2 imaging [72–74]. USG can be used for assessment of maxillary sinuses, but results are found to be inconsistent [75]. 

In acute cases, complete blood count, ESR, and blood cultures provide useful data. Tests for allergy, immunodeficiency, cystic fibrosis, and immotile cilia syndrome assist to detect associated conditions [76–78]. In AFS, total serum IgE and skin or *in vitro* tests for fungi and common allergens are usually positive [79, 80]. For collection of specimen for culture from the maxillary sinus, a maxillary sinus puncture is the standard criterion. However, it is painful, needs patient cooperation, and can be done only under anaesthesia in children. Culture swabs obtained from middle meatus or anterior middle turbinate correlate well with cultures from maxillary or ethmoid sinuses. However, random nasal swab cultures bear no correlation with maxillary sinus cultures. Gram-staining, aerobic and anaerobic cultures, and fungal cultures can be performed on the collected swabs to guide appropriate antimicrobial therapy. Flexible endoscopes are also employed for such specimen collections. Biopsy taken during endoscopic sinus procedures shows submucosal inflammatory infiltrates in acute and chronic rhinosinusitis. In AFS, eosinophils and Charcot-Leyden crystals predominate [81]. A positive fungal culture grown from infected sinuses is not confirmative of allergic fungal sinusitis. The isolated fungi may be a saprophyte, or the method of specimen collection and handling may also influence the yield. Presence of allergic mucin is a reliable pointer towards the disease [82]. 

## 7. Management

### 7.1. Conservative

An acute attack of rhinosinusitis is usually self-limiting and recovers with symptomatic treatment and with minimal intervention. Steam inhalation, adequate hydration, instillation of topical decongestants, warm facial packs application, and saline nasal drops are useful. Elevation of head while sleeping gives relief. The nasal decongestants decrease mucus production and can be safely used for 5–7 days. Extended use beyond this period may lead to rebound vasodilatation and worsening of nasal stuffiness [83]. The study by McCormick et al., however, did not show any benefit of topical decongestant with oral antihistamine in children with acute rhinosinusitis [84]. Nasal saline irrigations, nasal steroids, and topical cromolyn have been found to be useful. The saline irrigations assist to mechanically clear secretions, minimize bacterial and allergen burden, and improve mucociliary function [85]. Nasal steroidal or cromolyn drops or sprays improve symptoms in children with concurrent nasal allergy. Short burst of systemic steroids is employed preoperatively to minimize intraoperative blood loss in children with nasal polyp [86]. Antihistamines are beneficial in those with associated nasal allergy. But they have a tendency to inspissate the secretions and further worsen rhinitis and ostial obstruction. Mucolytics have been noticed to have variable effects. No proper randomized and controlled studies have been performed to evaluate their efficacy in such patients [87, 88]. Environmental pollutants worsen the situation, and hence avoidance of them tends to improve rhinosinusitis [89]. Antibiotics are usually not warranted. A “wait-and-watch” policy for 7–10 days is fruitful and cost-effective. About 90% recover without antibiotics in a week [90]. Antibiotics are reserved for children with severe acute sinusitis, toxic features, suspected complications, or persistence of symptoms [91]. Choice of antibiotics should be guided by local susceptibility studies, safety profile, and child's age. Usual preferred are amoxicillin, coamoxiclav, oral cephalosporins, and macrolide group of antibiotics. 2 weeks course is usually required [92]. Associated conditions should be simultaneously and individually addressed as follows.

1. Respiratory allergy. Allergen avoidance, environmental control, topical nasal steroids, second-generation antihistamine, leukotriene receptor antagonist, and immunotherapy are common measures to control allergic rhinitis. Immunotherapy is valuable for children with known allergens that cannot be avoided and where conservative therapy has not been advantageous. Anti-IgE therapy has been found to provide clinical benefit in patients with seasonal allergic rhinitis [93, 94]. Inhaled bronchodilators and optimal use of steroids could control bronchial asthma. Removal of trigger factors from the environment or diet also aid in minimizing asthmatic attacks. Active and/or passive smoking should be curtailed.
2. Gastroesophageal reflux. Elevation of the head end of bed, small, frequent and thickened feeds, avoiding near-bedtime feeds, H2-blockers, prokinetic agents, and hydrogen ion pump inhibitors are used to control reflux [95].
3. Cystic fibrosis. Nasal irrigations, nasal steroids, antibiotic courses, nebulized antibiotics, chest physiotherapy, and exercises aid to clear the copious secretions and thwart infections [96].
4. Immunodeficiencies. Aggressive treatment of recurrent infections and regular immunoglobulin infusions could control secondary infections in such patients [97].
5. Immotile cilia syndrome. This requires vigorous removal of secretions which in turn causes a decline in infection rate and associated complications [98].
6. Removal or correction of nasal obstructions.

Since CRS harbours extended-spectrum *β*-lactamase- (ESBL-) producing multi-drug-resistant polymicrobes, a broad-spectrum *β*-lactamase-stable second-line antibiotic is preferred as the first choice [99]. Antibiotics with activity against aerobic and anaerobic ESBL-producing bacteria and group A *β*-hemolytic streptococci (GABHS) will be able to provide clinical and microbiological clearance. Lincomycin, clindamycin, coamoxiclav, metronidazole with a macrolide, fluoroquinolone, aminoglycoside, expanded-spectrum cephalosporins, or carbapenems are usually effective [7, 100]. The therapy could be later deescalated based on susceptibility tests. This lessens chances of increasing resistance. Cefuroxime, cefpodoxime, cefdinir, vancomycin, clindamycin, and antipseudomonal antibiotics are all useful for polymicrobial infections. Parenteral therapy may be required in severe resistant cases [101]. Though there is no grade 1 evidence to support antibiotic usage in CRS in children, antibiotics are often used to treat CRS in children. Duration of therapy is for 3–6 weeks. Improvement in symptoms was seen after 22.35 ± 5.04 days (mean) of antibiotic treatment [102, 103]. The role of long-term intravenous antibiotics for CRS is not universally established, though a subset of CRS may benefit from such therapy [104]. Topical antibiotics have also been tried with varied results. Various modes of drug delivery are employed with ongoing research to enhance the drug delivery and deposition into the sinuses [105]. Prolonged low-dose oral antibiotics were found to be beneficial for CRS, but more studies are needed before recommending it as standard treatment [106]. Saline nasal irrigation is useful as adjuvant therapy in CRS and is found to be well tolerated in children [107, 108]. Since biofilm formation is the rule in CRS, modulation of the matrix and novel methods of biofilm disruption may be tried [109–113]. Drugs targeted against production or action of the AMCase enzyme could be fruitful in the management of persistent and recurrent sinus infections [43]. Oral enzymes such as rutin and bromelain are useful as adjunctive therapy [114]. Some traditional ayurvedic decoctions such as *Pitawakka Navaya *are relatively safe and useful as supplementary treatment in chronic sinusitis [115]. In AFS, it is difficult to remove the thick fungal debris and mucin in the sinal cavity. Surgical cleansing, antifungal medicines, steroids, and immunotherapeutic measures are used to achieve eradication with variable success [116]. Followups of cases with immunotherapy showed no recurrence after 7–17 months [117].

### 7.2. Surgical

Adenoidectomy is usually the first surgical intervention considered for children with CRS. It removes the obstruction as well nidus of infection [118–121]. A risk-benefit analysis should be carried out before considering other surgical approaches in children. There has been major transformation in diagnosis and therapy of rhinosinusitis due to the technical advances in medical endoscopes. When appropriate, maximal medical therapy fails or with associated anatomic aberrations, surgical interventions are contemplated in rhinosinusitis. About 2/3rd of patients of CRS fail maximal medical therapy and need to go ahead with surgery [122]. Almost universally, children with diagnosis of AFS need operative intervention, postoperative medical management, and close long-term monitoring and followup [123]. Surgery aims to restore sinus ventilation and ciliary function. Functional endoscopic sinus surgery (FESS) is done via a flexible endoscope and is beneficial for management of chronic sinusitis. However, in children, it is reserved only for complicated cases. FESS can remove the thick tenacious secretions, debris, and mucin in allergic fungal sinusitis which are otherwise difficult without open surgical methods. Detergent, 1% solution in normal saline of Johnson and Johnson baby shampoo, topical antibiotics such as gentamycin/tobramycin or antifungal drugs, and tea or sinufresh could be used during surgery to irrigate the sinus and improve outcomes [124–127] Baby shampoo contains a zwitterionic surfactant, which disrupts the biofilm. This could also be used for nasal irrigation post surgery [126]. Naraghi et al. found that the quality of life was improved significantly in patients after FESS [128]. FESS should be done with great care especially in those with AFS because in them bone dissolution is common and there is a potential risk of inadvertent penetration into orbit or cranium [129]. Animal studies have shown that even limited surgeries could affect sinus and facial growths. Hence FESS in children should be considered only after weighing the pros and cons of the procedure [130]. 

Other more invasive surgical techniques for maxillary sinusitis include antral washout, natural ostotomy, intranasal inferior meatal antrostomy (INA, nasoantral window), middle meatal antrostomy, Caldwell-Luc's operation (intraoral maxillary antrostomy), and uncinectomy (with or without endoscope and with or without maxillary antrostomy). All of these operations have their own indications, limitations, and problems [131–133]. Natural ostotomy and INA have been compared in rabbits and found to have similar outcome results [134]. Such comparisons unfortunately have not been studied in humans or children. Endoscopic middle meatal antrostomy has been shown to be better for maxillary sinusitis compared to the Caldwell-Luc operation [131]. Mycetomas and fungal balls in chronic rhinosinusitis also often require surgical debridement [135]. Intranasal antifungals have been studied and found to be of benefit for fungal chronic sinusitis; however, Cochrane analysis has shown no significant usefulness of topical or systemic antifungals over placebo for chronic sinusitis [136, 137]. Balloon sinuplasty is a new procedure in which ostia are dilated with the help of balloons. It could be tried before FESS for those who fail to respond to medical treatment and have minimal anatomic findings on CT scan [138]. A comparative outcome analysis of FESS alone versus balloon catheter sinuplasty in pediatric CRS revealed that both have similar outcomes, but antibiotics were required significantly lesser in the balloon catheter sinuplasty group [139]. 

## 8. Management of Complications

Intraorbital and intracranial complications are common in chronic sinusitis, and fungal sinusitis and with cystic fibrosis and immunodeficient states [140, 141]. Meningitis, abscess, and cavernous sinus thrombosis may occur. Sinusitis may extend to adjacent tissues and cause adenoiditis, serous or purulent otitis media, laryngitis, and dacryocystitis. Osteomyelitis and mucocele formation are also noted. Hospitalization and intravenous antibiotics may be required for treatment of these complications. Resistance rates are higher in complicated cases and culture-targeted antimicrobial therapy may be more beneficial for early resolution of symptoms. Prolonged course of antibiotics for 4–6 weeks may be necessitated in them. Cerebral venous thrombosis needs anticoagulation [142]. Nasal decongestants and steroids and nasal saline irrigation may be required for a longer time in such patients even after cessation of antimicrobial therapy. Pollutants, irritants, and allergens in the environment increase symptoms and avoidance of them is of benefit. Specific immunotherapy will be advantageous in cases where allergens cannot be avoided. Aggressive management of acute attacks will preserve mucosal integrity and ciliary function. Dental management for odontogenic sinusitis should be performed and children with sinusitis-associated HSP should be managed appropriately [143, 144]. 

## 9. Prognosis

Prognosis depends on stage of rhinosinusitis, associated conditions, rate of complications, type and severity of infection, host factors, environmental factors, compliance with treatment, and treatment modality employed [145]. Acute rhinosinusitis if managed well has potential to recover with no sequelae. Recurrence rate would be lower if associated conditions are rectified simultaneously and negative environmental factors are eliminated. Once CRS sets in, prognosis is guarded. Work productivity and quality of life are lessened. Early and aggressive treatment could give satisfactory outcomes and avert complications. 

## 10. Prevention

As a general rule, prevention of risk factors can help avoid development of rhinosinusitis. These include environmental pollutants including tobacco smoke, repeated colds and upper airway infections, daycare centre attendance, nasal allergies, and anatomical aberrations. These should be managed on a war footing in order to avoid their development into rhinosinusitis. Acute attacks of rhinosinusitis should be optimally managed to prevent progress to chronicity [146]. Prevention of adhesion and inhibition of quorum signalling may diminish biofilm formation and its associated problems of nonresponse to medical line of treatment [147]. Influenza and pneumococcal vaccines could also lead to fall in upper airway infections and hence rhinosinusitis [148]. Swimming in pools with high chlorine content may also worsen mucosal swelling and lining. Hence care should be taken at such places. Frequent plane flyers may also see worsening of symptoms with flights and precautions during such flights may help [149, 150]. 

## 11. Conclusions

Rhinosinusitis is an upper airway infection with chronic implications. Prompt management of acute cases would prevent cases slipping into chronicity with resistant polymicrobial infections. Management of chronic rhinosinusitis is an expensive, long-term affair with high likelihood of complications. Hence prevention and control of rhinosinusitis will assist in decreasing morbidity and lessen the burden on healthcare expenditure. Achieving sinonasal eutrophism and efficient mucociliary transport is the keystone to sinus health and reduction of recurrences.

## Figures and Tables

**Figure 1:**